# *“There’s a housing crisis going on in Sydney for Aboriginal people”*: focus group accounts of housing and perceived associations with health

**DOI:** 10.1186/s12889-016-3049-2

**Published:** 2016-05-24

**Authors:** Melanie J. Andersen, Anna B. Williamson, Peter Fernando, Sally Redman, Frank Vincent

**Affiliations:** School of Public Health and Community Medicine, The University of New South Wales, Sydney, Australia; The Sax Institute, 235 Jones St, Haymarket, 2007 Australia; The Aboriginal Medical Service Western Sydney, 2 Palmerston Rd, Mt Druitt Village, 2770 Australia

**Keywords:** Aboriginal, Indigenous, Housing, Urban, Focus groups, Social determinants of health, Framework analysis

## Abstract

**Background:**

Poor housing is widely cited as an important determinant of the poor health status of Aboriginal Australians, as for indigenous peoples in other wealthy nations with histories of colonisation such as Canada, the United States of America and New Zealand. While the majority of Aboriginal Australians live in urban areas, most research into housing and its relationship with health has been conducted with those living in remote communities. This study explores the views of Aboriginal people living in Western Sydney about their housing circumstances and what relationships, if any, they perceive between housing and health.

**Methods:**

Four focus groups were conducted with clients and staff of an Aboriginal community-controlled health service in Western Sydney (*n* = 38). Inductive, thematic analysis was conducted using framework data management methods in NVivo10.

**Results:**

Five high-level themes were derived: the battle to access housing; secondary homelessness; overcrowding; poor dwelling conditions; and housing as a key determinant of health. Participants associated their challenging housing experiences with poor physical health and poor social and emotional wellbeing. Housing issues were said to affect people differently across the life course; participants expressed particular concern that poor housing was harming the health and developmental trajectories of many urban Aboriginal children.

**Conclusions:**

Housing was perceived as a pivotal determinant of health and wellbeing that either facilitates or hinders prospects for full and healthy lives. Many of the specific health concerns participants attributed to poor housing echo existing epidemiological research findings. These findings suggest that housing may be a key intervention point for improving the health of urban Aboriginal Australians.

## Background

Poor housing can affect health directly and indirectly and can have both short and long term health impacts [1–3]. Housing is often named as a key determinant of the health and life expectancy gap between Aboriginal and Torres Strait Islander Australians (hereafter Aboriginal) and non-Aboriginal Australians [4]. ‘Healthy Homes’ are one of seven action areas in the Coalition of Australian Governments' *'Closing the Gap’* Campaign [5], a recognition both of Aboriginal housing disadvantage and of the growing body of international evidence about the associations between housing conditions and human health [1, 6, 7].

Studies in remote Aboriginal communities in Australia have found major problems with housing quality and availability [8, 9]. Associations have been demonstrated between poor remote housing and specific health problems, e.g. poor overall functional condition of housing and respiratory infection [10, 11]. Studies of the health of Aboriginal children living across urban, regional and remote areas have found associations between reported housing problems and ear, skin and chest infections [12, 13]. Similar housing and health problems have been documented amongst indigenous peoples living in Canada, North America and New Zealand [14–16].

As is the case with Aboriginal health research in Australia [17, 18], the majority of Aboriginal housing research and policy has focussed on Aboriginal people in remote communities [6, 19]. However, over 70 % of Aboriginal Australians live in urban areas or major regional centres [4] and 60 % of the burden of illness amongst Aboriginal people is accounted for by those living outside remote areas [20]. The data available suggests urban Aboriginal households also experience significant housing disadvantage and they are more likely to live in unaffordable housing than those in remote areas [19]. A qualitative study with Aboriginal people in Perth and regional Western Australia described housing careers characterised by poverty, difficulty accessing affordable housing, racism, insufficient social housing, difficulty navigating the social housing system, overcrowding, forced evictions and insecure tenure [21]. Aboriginal leaders have called for greater recognition of the housing needs of urban Aboriginal people [22, 23], yet direct research and policy activity in this space remains limited [19, 24, 25].

The current study examines the housing experiences of Aboriginal people living in Western Sydney. It is part of the Study of Environment on Aboriginal Resilience and Child Health (SEARCH), a cohort study of 1482 urban Aboriginal children in New South Wales, Australia [26]. Housing is a focus area in SEARCH, having been nominated by urban Aboriginal community leaders as a key concern in relation to health. SEARCH is the result of a long-term collaboration between the Aboriginal Health and Medical Research Council of New South Wales, the Sax Institute, University of Sydney, Australian National University, Sydney Children’s Hospital Network, policy and program agencies and four Aboriginal community-controlled health services (ACCHS) located in Western Sydney, South Western Sydney, Newcastle and Wagga Wagga.

## Methods

### Setting

This research took place at the Aboriginal Medical Service Western Sydney (AMSWS) in the Blacktown Local Government Area of Sydney, Australia. This residential area, 40km west of the Sydney Central Business District, is home to more urban Aboriginal Australians than anywhere else in Australia, approximately 31% of the total urban New South Wales (NSW) Aboriginal population [19, 27]. Western Sydney is classified as a disadvantaged area with high rates of unemployment and public housing and low educational attainment and incomes [28, 29]. The majority of dwellings are detached houses, with some semi-detached houses and relatively few apartment blocks [27].

### Ethics

This study was approved by the AMSWS, the Aboriginal Health and Medical Research Council (686/09) and the University of New South Wales (10083). All data collected as part of SEARCH is owned by the participating health service. Focus group participants were provided with participant information sheets and verbal explanation was given about the study purpose, the voluntary nature of participation, confidentiality procedures and how data would be recorded and used prior to consent forms being signed.

### Study design

Focus groups were used to capture the breadth and richness of community views. They provide a culturally appropriate social space for building on ideas and discovering agreement or disagreement on a topic [30]. In this setting it is possible both to discover social norms and explore variation and complexity in views [31].

### Participant selection

Participants were purposively selected to include people of particular ages, genders, life stages, health and socioeconomic circumstances. After discussions with FV, CEO of the AMSWS, and other key members of staff, groups were formed based on the clinical services provided in order to recruit relevant groups while creating a degree of homogeneity to help participants feel comfortable. Staff and clients were invited to participate by the team leaders of each targeted service. Four groups were held:

- child and family (*n* = 12), 6 staff, 4 young mothers, 1 father and 1 grandmother
- chronic care (*n* = 9), 1 staff and 8 older men (2) and women (6) with chronic health issues
- social and emotional wellbeing (*n* = 11), 5 staff (4 male, 1 female) and 6 clients (4 female and 2 male, ages ranging from 20-60 years)
- staff (*n* = 11), 6 female, 4 male. Five staff had also attended the group relevant to their clinical speciality.

Thirty five of the thirty eight participants were Aboriginal. Participants had a range of levels of education and differed in terms of employment and housing status. The mix of staff and client participants in the groups occurred organically and reflects the relative lack of division between these social groups in many Aboriginal communities, as compared with the distinction normally observed in mainstream health services.

### Research process

Focus groups were facilitated by MA (female Caucasian PhD candidate with a health background) and PF (male Aboriginal researcher with a background in community-controlled health service provision) in late 2010. Three broad trigger questions were asked: Are Aboriginal people in Western Sydney having problems concerning housing? If so, what sorts of problems? What sort of effects are housing issues having on people? A conversational space was created where participants discussed housing issues of concern to them and facilitators probed for detail [32]. Through this process additional domains were identified and explored. Groups ranged from 62 – 150 minutes in duration.

### Analysis

Dialogue was recorded and transcribed verbatim. Transcripts were coded manually by MA using open coding techniques from a realist stance. AW conducted independent open coding on a transcript; the codes and thematic categories derived were very similar. Where minor conceptual differences arose, they were resolved through discussion*.* The codes were organised into a conceptual framework, with higher-level themes and an index of subthemes. PF reviewed these initial analyses. A community feedback session at the AMSWS was held in August of 2011 and attended by 4 Aboriginal focus group participants, 2 staff and 2 clients. Feedback was positive; some suggestions for additional inclusions and the prioritisation of certain themes over others were made, but no suggestions of omissions or misinterpretation.

Deeper analysis was then conducted using the Framework method, a case and theme-based approach to data management, in NVivo 10 (QSR International, 2010). Framework matrices enabled clear visualisation of the data and facilitated analyses of associations between themes, and of the variation and agreement between and within focus group cases. COREQ guidelines for reporting qualitative studies have been followed. Explanatory accounts have mostly been limited to the explicit reasons for phenomenon given by participants [31].

## Results

The majority of participants (20) lived in state-owned and managed public housing. Of these, nine were in mainstream public housing managed through Housing NSW. Eleven lived in state-owned and managed Indigenous housing (SOMIH), housing allocated exclusively to Aboriginal people through the Aboriginal Housing Office (AHO). Two lived in Aboriginal community housing owned by the local Aboriginal Land Council. Five participants lived in privately rented homes (4/5 were AMSWS staff, 1/5 a client). Four participants were homeless (2/4 staying with family or friends, 2/4 in emergency accommodation provided by the state), two participants had a mortgage (both staff, one Aboriginal and one non-Aboriginal). Five participants did not specify their housing situation. AMSWS staff participants were embedded in the community. Most lived locally, had experience of living in social housing themselves and regularly assisted their clients with housing issues. These experiences, combined with their health knowledge, made them key informants in this study. There was a high level of agreement between the views of AMSWS staff and clients, hence findings have been combined and presented thematically.

Five high-level themes were derived from the data: the battle to access housing; secondary homelessness; overcrowding; the poor condition of available housing; housing as a crucial determinant of health across the life course. Participants also discussed broader contextual issues surrounding housing problems, detailed in a separate paper.

### The battle to access housing

Participants indicated that most Aboriginal people living in Sydney had limited housing options, with housing affordability described as a constant and pressing concern for many. Home ownership was described as unfeasible for most of the Aboriginal community and rarely discussed by participants. Sydney’s private rental market was also considered inaccessible to many Aboriginal people, particularly young people, due to prohibitive costs and/or their uncompetitive tenancy or work histories. Discrimination from real estate agents and landlords was repeatedly described as another key barrier*.* Some participants recounted being falsely told there were no rental properties available, others submitted countless unsuccessful applications.

*“to get a rental house – it's almost impossible for an Aboriginal person… there's proof of income, there's good tenancy records… you have got to compete with about 30 or 40 other people …. in all reality, except for black housing and subsidised NSW Housing, you wouldn't have a house, you just wouldn't have a house”*

*Middle-aged male AMSWS staff*

Participants said social housing was the only option for much of the Aboriginal community. However, social housing was described as hard to access, with waiting periods of up to 15 years reported. Homes owned by the local Aboriginal Land Council were also said to be in short supply. This chronic shortage of affordable housing also meant people felt unable to insist that their housing met basic standards. Some also lived in housing that was inappropriate for their needs, for example frail aged people living up several flights of stairs.

*“that's why they put up with sub-standard housing … because they've got nowhere else to go…. when you're vulnerable, you get it and that's it. If you had money, you wouldn't be putting up with it"*

*Middle-aged female AMSWS staff*

### Secondary homelessness

While primary homelessness (rooflessness) was not described as common amongst Aboriginal people in Western Sydney, secondary homelessness (transient or emergency accommodation) was. Homelessness was mostly attributed to the long wait for social housing or forced evictions, often due to falling behind in rental payments. Participants said some people experiencing homelessness were eligible to stay in temporary state-provided accommodation, including low-cost motels, caravan parks or boarding houses. However this was described as incredibly stressful, often involving frequent moving between poorly located placements (no transport, services) that were often described as unsuitable for children,

*“She can't take baby to the doctors, she can't go to the shops to get milk if she needs it. Some of the places that they're putting the Mums haven't got cooking facilities… she's got a young baby, and she can't even warm up a bottle of milk, and that's where a lot of them are” Young female AMSWS staff*

Participants reported that most Aboriginal people instead rely on their social networks to avoid primary homelessness, often living with family and friends for extended periods of time.

*“One of the family members will get a house, and because we are very family-orientated, you won’t leave family on the street, we'd all rather pack in” Middle-aged female client*

Participants said staying with others often entailed moving from house to house *(“house-hopping”*), sometimes with children in tow. One participant and her four children had been hopping for six years while awaiting social housing and applying unsuccessfully for private rental properties. Participants of both genders in all focus groups said this was common,

*“A lot of our young Mums are like that. They have babies, they're house hopping… going from family to family to family. The babies aren't settled and the young mums aren't settled… it's just no good.”*

*Middle-aged female AMSWS staff*

Participants said even families with stable employment and housing were not ensured good living conditions, as they may be called upon to share their housing with extended family and friends who would otherwise be homeless. Older participants were more likely to have housing but many had extended family either living or staying with them. Those hosting people reported feeling anxious their neighbours may complain about occupancy levels or noise and those in social housing were worried they may be charged higher rent or evicted for breaking tenancy agreements. Some hypothesised that official measures of homelessness and overcrowding must underestimate the true scale of these problems, as many Aboriginal people are cautious about disclosing who lives with them for fear of these consequences.

### Overcrowding

*“Some of these families are living in overcrowded homes just beyond the ridiculous. Twenty people and more in a three bedroom place… because they just can't get housing” Middle-aged female AMSWS staff*

Participants reported that *“overcrowding is a big problem in a lot of the houses”*. The term *“overcrowding”* was spontaneously used by participants, not introduced by researchers. Several factors were believed to lead to overcrowding. Firstly, Aboriginal families are often large and there are insufficient affordable homes to appropriately accommodate them. Secondly, the community’s efforts to accommodate homeless family and friends often resulted in multi-family households,

*“my brother, he's living in a two bedroom and there's three families in there… they're sleeping on floors … Having that many people in a two bedroom, it kills you, you know?” Young male AMSWS staff*

Thirdly, participants said Aboriginal people were often called upon to host extended family who were visiting to access services, visit family and friends or attend community events.

Participants described households struggling to cope with insufficient access to space, privacy and basic amenities,

*“Imagine meal times, washing clothes, food preparation, all those things… we're forced to live in a communal situation… however the facilities are not there to cater for that” Middle-aged male AMSWS staff*

Participants said significant numbers of Aboriginal children in Western Sydney lived in overcrowded housing; many without adequate space to sleep, play or do homework. Overcrowded households were also described as interpersonally stressful environments, with people *“walking on eggshells to keep the peace*”*.* Participants believed overcrowding was inherently problematic, compounding other housing problems and a determinant of health in and of itself,

*“If you've got a house that's overcrowded, there’s a health issue. That's a health issue within itself. It's got nothing to do with the actual house” Young female AMS staff*

### The poor condition of available housing

*“All the houses here are inadequate, all the [state-owned public housing] homes are inadequate. Because they're that ancient, they don't get maintained properly…” Middle-aged female AMSWS staff*

Participants described the social housing in Western Sydney as generally old and in poor condition. They said the houses were often built from asbestos or other fibreboard, with poor insulation. Problems such as mould, damp, broken amenities, leaking rooves, structural problems, faulty plumbing and electrics, vermin infestation, and poor temperature control were reportedly common.

*“I could sit here for months and listen to stories that would horrify anybody” Older male AMSWS staff*

With few exceptions, participants said public housing tenants experience difficulty obtaining repair and maintenance services. When maintenance or repairs were done, the quality of the work was reportedly often poor, *“just Band Aid jobs” Young male client*

Homes owned by the local Aboriginal Land Council were generally described as being in reasonable condition. However, some participants indicated that Land Councils also had insufficient funds to provide good maintenance services. Most participants who rented their homes privately said their housing was not of a particularly high standard, despite being expensive.

### Housing a crucial determinant of health across the life course

*“There are major, major health problems associated with that housing” Older male AMSWS staff*

Participants repeatedly expressed the belief that housing problems negatively affected the physical health and social and emotional wellbeing of the Aboriginal community in Western Sydney. Housing was said to affect the health of *“the whole community here”*, though some health issues and their sequelae were said to manifest in different ways across the life course.

In regards to physical health, participants believed a key driver for high rates of communicable disease (namely cold and flu, gastroenteritis, ear, chest and skin infections) in their community was overcrowding,

*“In the overcrowded houses, if one of the kids gets sick the whole family gets sick” Young female AMSWS staff*

People associated mould and damp with the exacerbation of asthma and respiratory conditions. Injury risk posed by broken or faulty household fixtures was also a concern. Physical health problems were said to be of particular concern for children, the elderly and those with existing chronic health conditions.

In terms of social and emotional wellbeing, participants used words such as *“stressed”, “depressed”, “worried”*, “*frightened*” and *“terrified”* when describing housing problems. Housing was described as a pervasive source of stress affecting peoples’ lives daily. Some said the physical condition of their housing contributed to feelings of depression. Others reported feeling *“hopelessness”,* powerless to change their housing situation or that of people close to them. People believed housing problems, particularly secondary homelessness and overcrowding, placed strain on couple and family relationships, which in turn affected individual emotional wellbeing.

*"when you’re living around twenty people, your stresses are up and the mental illness comes along quite quickly” Young male AMSWS staff*

Participants considered childhood a time of peak vulnerability for poor housing to affect health. They described pathways by which poor housing affected the life trajectories of many Aboriginal children in Western Sydney. They said children who are regularly sick have patchy school attendance. Otitis media was emphasised as a housing-related illness of particular concern due to its prevalence and potential effects on hearing, speech, language, behaviour and education. Inadequate playing spaces were said to limit social and developmental opportunities for some. Frequent relocation, particularly due to homelessness, was said to be unsettling for children and to cause further disruption to schooling*.* Participants considered the effects of poor housing on child health and development as a key mechanism in the maintenance of generational disadvantage,

*“How can your kids move on and build a life? And change or break that cycle?… They’re set up to fail from the beginning” Elderly female client*

Parents, especially young and sole parents, were another group for whom housing issues were said to cause significant health and wellbeing problems. Participants said they were disproportionately exposed to chronic and pervasive stress, particularly those unable to access stable housing,

*“… and that is obviously impacting on [client’s] emotional state, and her child, and that is having a great deal of impact on health. Not being able to get decent accommodation, worrying about it all the time” Middle-aged female AMSWS staff*

Participants said precarious housing was disempowering, making it more difficult for young parents to gain employment or complete higher education, in turn making it harder to secure decent housing. Secondary homelessness, house-hopping, overcrowding and even poor dwelling conditions were seen to make parenting difficult in multiple ways including the ability to: store and cook nutritious food; get children to school and medical appointments; provide consistent parenting (e.g. comfort or discipline); and keep children safe from various forms of harm, particularly in households where they were *"not the boss"*. These difficulties were said to heighten stress and have further implications for child health. Staff expressed frustration that while recent health campaigns meant parents and carers were often knowledgeable about the value of healthy environments for their children, many were unable to control their home environment,

*“I think that housing is one of the major issues for these families in keeping the children safe, having appropriate housing” Middle-aged female AMSWS staff*

Participants said living in crowded, stressful households sometimes meant older people were less able to attend to their own health needs. Older participants believed that both their current housing and poor housing during their childhood affected their health, particularly through the exacerbation of chronic illnesses now being experienced. They also expressed strong concern about the damage they believed poor housing was doing to the health of new generations of Aboriginal children,

*“It's affected our health, and it's gonna affect our kids' health - you can see it now with our kids that have got kids, the problems they're having. We wonder what's going to happen to them and what's going to happen to their kids in housing?” Elderly female client*

Participants regarded the link between housing and health as common sense. They considered housing a crucial and under-resourced determinant of health,

*“We are covering health, we are covering education… but it's housing that's just lagging far, far behind and until they address that one, you know, it’s…” (holds up hands) Middle aged female AMSWS staff*

Participants asserted that while so many Aboriginal people experience the housing problems described, the health, education and employment gaps between Aboriginal and non-Aboriginal Australians would remain,

*“Because housing affects the rest of your life… it’s so important”*

*Middle-aged female AMSWS staff (many say “yeah”)*

## Discussion

While this study is not the first to call attention to the unmet housing needs of urban Aboriginal Australians [6, 19, 21, 33], these findings add new insights into how Aboriginal people in this disadvantaged part of Sydney perceive their housing situations. Housing was described as a pivotal and far-reaching determinant of health for Aboriginal people in Western Sydney and a key mechanism for the maintenance of intergenerational disadvantage. Participants were particularly concerned about the poor living conditions of children and the impact they have on health and developmental trajectories. While young people and families were said to be most likely to experience difficulty securing appropriate housing, the burden of this difficulty was spread across the life course and to some extent also across the socioeconomic spectrum, as those with more secure housing were called upon to assist extended family and friends experiencing hardship.

The extreme difficulty participants experienced when trying to access housing is perhaps unsurprising given Sydney’s current housing landscape. Housing NSW acknowledges *“a shortage of suitable accommodation for local communities in most areas”* [34]. Expected waiting times for social housing in most Western Sydney suburbs were listed as *“10+ years”* for general applicants at the time of writing [35]. A recent audit of available rental properties in Sydney, including outer Western Sydney, found that almost none were affordable for low income households [36]. In the current study, difficulty accessing suitable housing was reported to be exacerbated for Aboriginal people due to discrimination from private housing providers. Evidence of racial profiling affecting housing opportunities has been found in Australia and overseas [37–40].

Participants spoke emphatically about the health and social problems they associated with “overcrowding”. This is a noteworthy finding as the notion of overcrowding is controversial. Some suggest that the term 'overcrowding' is inappropriately laden with negative meaning as Aboriginal people may have a cultural preference for living in extended family households [41–43]. High household occupancy has even been associated with better emotional wellbeing in Aboriginal children in some remote communities [44]. Participants in this study, however, expressed a clear preference for living near but not with extended family, particularly as available housing is not designed for multi-family households. High household occupancy was considered inherently problematic, negatively affecting people’s health and wellbeing. This view is in keeping with research, overseas and in remote Australia, that demonstrates significant associations between high household occupancy and health problems, particularly infectious disease [45–47]. Participants felt the combination of housing unaffordability, homelessness and kinship obligations were the main drivers for overcrowding in Aboriginal households, a relationship which has been documented elsewhere [48]. Both the value of such social capital [49] as protection against rooflessness and the high cost paid by hosts in crowded households have been noted previously [48].

The poor condition of the ageing social housing stock in New South Wales is also widely acknowledged, as are the maintenance affordability problems this poses for housing providers [50, 51]. Poor public housing conditions and difficulty getting required maintenance performed has also been reported in other urban parts of Australia [21, 52]. Another finding of note is that participants in this study invariably expressed the wish to obtain stable housing. They believed a key driver of homelessness for Aboriginal people in Western Sydney was the lack of accessible, affordable housing. This differs from the situation in remote communities, where it has been suggested that homelessness is driven by factors beyond inadequate housing supply such as the need for mobility to access services and significant places and cultural or personal factors [41].

A deep and broad knowledge of environmental health was evident in participants’ discussion of housing. Many of the specific links participants posited between housing and health echo existing epidemiological research findings [6, 7, 47]. Household crowding is associated with infectious disease, including otitis media [45]. Otitis media in turn is associated with hearing loss, speech and language problems in children [53]. Damp and mouldy houses are independently associated with asthma, other acute and chronic respiratory conditions, depression, anxiety and recurrent headaches [54]. Poor dwelling conditions also increase injury risk, particularly for children and the elderly [55]. Unstable housing tenure, particularly homelessness, has been shown to negatively affect physical and mental health, child development, and social and economic participation [2]. Moreover, the kind of chronic, pervasive stress participants described experiencing due to housing problems has been described as in the literature as "toxic" [56]. Such pervasive stress in childhood is associated with lasting health effects, including chronic disease and mental ill health [57]. Similarly, the kind of racism participants described experiencing is associated with psychological distress, mental ill health and poor physical health. Study participants also identified that children, young families, the elderly, those living in poverty and those with existing health conditions are most vulnerable to housing problems; that is they are most likely to experience housing problems and are most susceptible to the ill effects of poor housing [2, 3, 52, 58–60].

The detailed information given by participants and their ability to address current knowledge gaps or areas of controversy in the literature highlights the value of working with Aboriginal communities to identify problems, potential causal pathways and ultimately solutions to the sorts of complex problems with which they are intimately familiar. Their lived experiences unsurprisingly renders them experts in Aboriginal affairs [61]. Participants in this study expressed a holistic view of health, considering health to be intrinsically linked with environmental and social factors. The compatibility between Aboriginal conceptualisations of health and the social determinants of health has been noted elsewhere [62], as have the ethical, moral and practical imperatives of listening to Aboriginal voices [32, 61]. Qualitative research methods offer a particularly appropriate means of exploring and communicating Aboriginal knowledge and world views [32].

Participants of this study were unanimous in believing that if Australia is serious about ‘closing the gap’, more investment in Aboriginal housing, including urban public housing, is required. While federal and state governments have established a National Partnership Agreement on Remote Indigenous Housing and committed billions of dollars to improve remote housing [25], there is no comparable agreement on urban Indigenous housing. Instead the housing needs of urban Aboriginal people are addressed under mainstream social housing and homelessness agreements [24]. Study participants expressed the belief that housing problems in the Aboriginal community went beyond those experienced by other low income groups. Many additional systematic factors were said to affect housing prospects and conditions for Aboriginal people, including discrimination and cultural responsibilities to extended family. Along with the participants of this study, academic Nicholas Biddle warns that the closing the gap campaign will not be successful unless the issues facing city-dwelling Aboriginal people are specifically addressed,

*“To close the gaps, all levels of government will have to have one eye on remote Australia with the other on indigenous gaps in the cities”* [33]*.*

While housing may seem beyond the scope of the health sector, there is a long-standing relationship between public health and housing [63]. Public health professionals have an obvious role to play in describing the scale and health impacts of housing problems. Public health can also engage in a range of other activities to improve housing conditions, including advocacy and awareness raising, collaboration with the housing sector, the provision of direct services and evaluation of the effectiveness of housing improvement programs. For example, New South Wales Health (NSWH) have long conducted *‘Housing for Health’*, a housing intervention designed to improve aspects of housing known to affect health, chiefly for households in Aboriginal community-controlled housing in rural and remote areas [11]. Amongst other benefits, residents of households who received *Housing for Health* had 40% lower rates of hospital separation for infectious diseases than comparable rural and remote Aboriginal communities who did not receive the program [11]. *Housing for Health* was recently piloted with 44 Aboriginal households living in state-owned social housing in Western Sydney [64]. This involved a unique collaboration between the Western Sydney Public Health Unit, NSW Health Aboriginal Environmental Health Unit, Housing NSW, the NSW Land and Housing Corporation and the AMSWS [64]. This cross-sectoral collaboration, which was assisted by SEARCH researchers, took many years and much determination from the Western Sydney Public Health Unit and good will from all parties to negotiate, but may provide a viable model for the ongoing improvement of existing social housing conditions for urban Aboriginal households.

In New Zealand, a public health research group has produced a large body of high quality evidence demonstrating the relationships between housing and health and the cost-effectiveness of investment in housing improvement programs [65–67]. This evidence has been successfully used to lobby the government to fund widespread housing improvement programs and to influence public discourse about the importance of social housing stock as a key part of the nation’s infrastructure [67, 68]. This group has also developed an evidence-based, standardised housing assessment for use to determine if housing meets basic health, safety and energy efficiency standards [69]. Trials are underway to test the feasibility of making the obtainment of this “Warrant of Fitness” compulsory for homes leased in the private sector. The current study reports the views of clients and staff of the Aboriginal Medical Service Western Sydney. They are not necessarily reflective of the views of other Aboriginal people in Sydney or elsewhere. This study reports the housing and health issues that study participants were aware of and concerned about. It is not intended to comprehensively capture all potential pathways by which housing conditions may be materially affecting health and wellbeing. It may be useful to conduct groups with urban Aboriginal people who own or are paying off their own homes or those who are sleeping rough, as these groups may have a different range of housing experiences and perspectives.

## Conclusion

This study has several implications. Housing appears to be a major issue for urban Aboriginal people, meriting targeted research and policy attention. Further inquiry into urban housing conditions and their health association is also indicated. These findings will inform quantitative research to be conducted by the study team. At an individual level, health professionals and educators working in urban settings should consider discussing housing with Aboriginal clients as part of holistic service provision. More broadly, public health has played an important role in advocating for improved housing in remote Aboriginal communities. A similar public health approach to housing may also benefit the many Aboriginal Australians living in our cities and suburbs.

### Ethics approval and consent to participate

Ethics approval was obtained from the Aboriginal Health and Medical Research Council (686/09) and the University of New South Wales (10083). Written consent was provided by all participants.

### Availability of data and materials statement

Transcript data will not be made available as it contains information through which participants may be identified. The data is owned by the AMSWS.

## Abbreviations

AMSWS: Aboriginal Medical Service Western Sydney. NSW: New South Wales
